# A cross-sectional study of sensory-motor neuropsychological function among sewage plant and sewage net workers exposed to hydrogen sulphide when handling wastewater

**DOI:** 10.1093/annweh/wxad051

**Published:** 2023-09-23

**Authors:** Lars Ole Goffeng, Åse Dalseth Austigard, Kristin H Svendsen, Øivind Skare, Elin Einarsdottir, Lene Madsø, Kari Kulvik Heldal

**Affiliations:** National Institute of Occupational Health, Group for Work Psychology and Physiology, PO Box 5330 Majorstuen, N-0304 Oslo, Norway; Department of Industrial Economics and Technology Management, NTNU - Norwegian University of Science and Technology, PO Box 8900, Torgarden, N-7491 Trondheim, Norway; Working Environment Office, Trondheim Municipality, PO Box 2300, Torgarden, N-7004 Trondheim, Norway; Department of Industrial Economics and Technology Management, NTNU - Norwegian University of Science and Technology, PO Box 8900, Torgarden, N-7491 Trondheim, Norway; National Institute of Occupational Health, Group for Work Psychology and Physiology, PO Box 5330 Majorstuen, N-0304 Oslo, Norway; National Institute of Occupational Health, Group for Work Psychology and Physiology, PO Box 5330 Majorstuen, N-0304 Oslo, Norway; National Institute of Occupational Health, Group for Work Psychology and Physiology, PO Box 5330 Majorstuen, N-0304 Oslo, Norway; National Institute of Occupational Health, Group for Work Psychology and Physiology, PO Box 5330 Majorstuen, N-0304 Oslo, Norway

**Keywords:** exposure index, H_2_S, nervous system, neuropsychological tests, job-exposure matrix, sewage, wastewater workers

## Abstract

**Objectives:**

Workers at sewage treatment plants are exposed to a complex mixture of toxins, including hydrogen sulphide (H_2_S). An issue of concern among sewage workers, is possible *negative nervous system effects from low-level H*_*2*_*S exposure*. Empirical neuropsychological evidence indicates both that low-dose exposure to H_2_S exposure affects the nervous system, and the contrary, that such exposure may facilitate nervous system function, since H_2_S is an endogenously produced central nervous system (CNS) gasotransmitter. The aim of this study is to describe a possible association between the H_2_S component of the total exposure and long-term effects on neuropsychological motor function among wastewater workers.

**Methods:**

Workers (*N* = 138) treating wastewater in 6 sewage-treatment plants, or in the sewer net system participated in a cross-sectional study. H_2_S exposure was expressed in a dichotomous exposure variable defining currently H_2_S-exposed (*N* = 112) and unexposed referent workers (*N* = 26), and a variable defining a job-exposure matrix for long-term total typical workplace H_2_S exposure. The participants went through neuropsychological tests for hand coordination, reaction time (SRT), and balance, and completed questionnaires. Pearson chi-square test or independent samples *t*-test was used when comparing the currently H_2_S-exposed workers with the unexposed control group. Multiple linear regression was used to assess associations between the independent variables age, smoking and exposure variables, and the neuropsychological tests.

**Results:**

The analyses indicate increased SRT in the currently H_2_S-exposed group compared to controls (mean [SD] = 225.8 [29.9] versus 210.7 [26.3] ms, *P* = 0.019), and an association between increased SRT and current H_2_S-exposure in the total study sample (β = 14.7, *P* = 0.026, *R*^2^ = 0.06, *P* = 0.050). Blindfolded balance testing indicates a nonsignificant trend in the *total study sample*, of reduced balance in the highest versus lowest H_2_S total long-term exposure-index group (Sway area [mean {SD}, mm^2^: 702 [410] versus 581 [278]), and a significant association between total long-term H_2_S exposure and reduced balance *among smokers* (Sway area, mm^2^ [β = 38.7, *P* = 0.039], mean sway, mm [β = 0.3, *P* = 0.015]).

**Conclusion:**

The observed trends and associations may be due to exposure peaks in certain work operations and pinpoint the importance of minimizing and avoiding exposure peaks, also when H_2_S time-weighted average measurements do not exceed an occupational exposure limit of 5 ppm.

**What’s Important About This Paper?**

Few studies have assessed sewage workers’ motor function with neuropsychological testing in an exposure-based epidemiological design. A dose-dependent negative association between balance, when blindfolded, and long-term workplace hydrogen sulphide exposure is described in otherwise healthy sewage workers who smoke. The study highlights the importance of minimizing hydrogen sulphide exposure peaks, even when time-weighted average measurements do not exceed an occupational exposure limit.

## Introduction

Workers handling wastewater at sewage treatment plants are exposed to a mixture of microorganisms, microbial components, and toxic gases, including hydrogen sulphide (H_2_S) (Laitinen et al. 1994; Guidotti 1996; Rylander 1999). The exposure complexity gives rise to a broad range of symptoms, including central nervous system (CNS) symptoms (Melbostad et al. 1994; Douwes et al. 2001; Thorn and Beijer 2004; Heldal et al. 2010), complicating an isolated dose–effect relationship for *each of the exposures*, based on occupational studies alone (Farahat and Kishk 2010).

When inhaled, H_2_S passes efficiently through the respiratory tract, is distributed throughout the body via the pulmonary blood supply, and reduces the capacity of cells to utilize intracellular oxygen in energy production of the mitochondria, through binding to cytochrome oxidase (histotoxic hypoxia) (Klaassen and Amdur 2013). H_2_S does not compete with oxygen in binding to iron in haemoglobin of the red blood cells in sufficient quantities to inhibit the oxygen transport to bodily cells (Guidotti 1996, 2010). Although H_2_S promotes the formation of methaemoglobin from haemoglobin (Saeedi and Najibi 2015), and oxygen does not bind to methaemoglobin, this hardly affects oxygen transport, since the amount of intracellular methaemoglobin compared to haemoglobin is <1% only (Umbreit 2007).

An exposure pattern with sudden *short-duration high peak exposures* to H_2_S, may be extremely hazardous. Acute high-dose H_2_S exposure (>300 ppm) can cause pulmonary oedema, and concentrations higher than 500 ppm may lead to immediate unconsciousness, so-called “knockdowns.” Single-exposure concentrations above 1,000 ppm (0.1%) H_2_S, may cause respiratory arrest and death (Svendsen 2001).

The histotoxic hypoxia is considered a too slow mechanism to account for the “knockdown” phenomenon and respiratory arrest, and sulphide is assumed to act on the respiratory drive also through mechanisms that affect brainstem networks (Svendsen 2001). Such toxicity is driven by concentration rather than duration, of exposure (Guidotti 1996; Woodall et al. 2005).

H_2_S may reach hazardous concentrations close to the ground or floor as it has a higher density than air (Rumbeiha et al. 2016) and may have acute toxic concentrations in breathing air in confined spaces, rooms, or tanks. Injury mechanisms may be hypoxia due to CNS-associated breathing stop, or traumatic brain injury, if falling after a sudden knockdown.

CNS cortical occipital and parietal areas, subcortical white matter, basal ganglia, including globus pallidus (Tvedt et al. 1991), putamen and caudate nucleus (Tvedt et al. 1989), brain stem (Reiffenstein et al. 1992), and Purkinje cells of the cerebellum, have shown to be susceptible to hypoxic injury after exposure to H_2_S (Lund and Wieland 1966; Guidotti 2010). These parts of the nervous system are associated with basic autonomic activity such as respiration, as well as body balance, motor functions, and coordination. Outcomes associated with balance have been observed in case studies where H_2_S exposure has led to unconsciousness (Tvedt et al. 1989; Kilburn 2003).

A concern among sewage plant workers or wastewater workers, has been possible negative health effects also from *low-level* H_2_S exposure. Case reports have indicated that repeated exposure to slightly elevated average concentrations of H_2_S (5–10 ppm) in the work environment (Tvedt et al. 1991; Richardson 1995; Watt et al. 1997; Lee et al. 2007; Farahat and Kishk 2010), increase the risk for developing chronic nervous system symptoms, like fatigue, headache, irritability, concentration difficulties, poor memory, and dizziness (Lee et al. 2007; Lewis and Copley 2015; Lim et al. 2016). However, it has been difficult to separate H_2_S effects from other co-exposures, and direct H_2_S effects from permanent long-term hypoxic effects following acute knockdowns with unconsciousness, since sewage workers may have experienced both.

Neuropsychological testing has been used to assess cognitive or sensory/motor problems among sewage workers, and at lower exposure levels, in the general public (Lewis and Copley 2015; Lim et al. 2016). The widely cited Kilburn studies include neuropsychological testing (Kilburn and Warshaw 1995, 1997, 2003, 2010), and conclude with considerable cognitive reduction among H_2_S-exposed study participants. However, the studies have been criticized because of symptom-based inclusion into the studies, inclusion of participants involved in litigation processes, and incorporating acute reactions into the exposure assessment (Guidotti 2010). One workplace study only includes neuropsychological testing of sewage workers with study inclusion based on H_2_S exposure rather than manifest symptoms (Farahat and Kishk 2010). That cross-sectional study, however, also includes exposure mixtures. Although Kilburn’s case series of highly exposed workers who were rendered unconscious, provides plausible information regarding the nature of the symptoms, his community study concluding with similar symptoms after long-term, low-level H_2_S exposure, has not been reproduced (Kilburn et al. 2010).

Two other community-based population studies of CNS effects after ambient low-dose background H_2_S exposure, lower than in occupational samples (Inserra et al. 2004; Reed et al. 2014), and an experimental chamber study of H_2_S exposure (Fiedler et al. 2008), even indicate marginally *better* neurobehavioral performance, in H_2_S-exposed groups compared to controls, most evident with tests of psychomotor speed, reaction time, manual motor dexterity, and sway/balance. This may be in line with a hypothesis that a very low exposure to H_2_S might stimulate CNS function (Reed et al. 2014), at least when exposure is in the range 0.5–5 ppm (Fiedler et al. 2008), 0–64 ppb (0–0.064 ppm) (Reed et al. 2014), or close to 0.09 ppm (Inserra et al. 2004). Since H_2_S is also an endogenously produced neurotransmitter, that at physiological or pharmacological levels may be beneficiary for the nervous system (Abe and Kimura 1996; Kimura 2011), this could be reasonable. However, such effects are dose-dependent (Zhang et al. 2017).

Thus, empirical evidence indicates both that low-dose exposure to H_2_S may have subtle negative neuropsychological effects and that such exposure may facilitate nervous system function. This unresolved issue constitutes a challenge in the dissemination of information of health risk and in preventive work among sewage workers.

In the study of health effects after long-term low-dose exposure, measuring critical aspects of occupational H_2_S exposure is challenging, due to the unpredictable and sudden nature of peak exposure episodes. An exposure index based on a job-exposure matrix (JEM), combining general concentration, exposure peaks, duration, and work tasks, is an approach to the study of possible long-term health effects after typical workplace exposure in groups of workers (Austigard et al. 2018), provided it catches the critical and relevant exposure factors (De Fruyt et al. 1998), and that long-term effects are not overruled by acute effects from recent or ongoing exposure.

Co-exposures not directly related to the work situation, may also influence the association between a critical occupational exposure and health effects. For instance, *upright body balance* is maintained by continuous motor and sensory feedback that involves and integrates impulses to and from several parts of the nervous system, including visual, proprioceptive, and vestibular control mechanisms (Morton and Bastian 2004; Qiao et al. 2021) and is thus a whole-brain phenomenon. Not only are the brainstem, cerebellum with cerebellar Purkinje cells, the basal ganglia, midbrain/mesencephalon, or thalamus involved, but even the spinal cord and cortical structures (Morton and Bastian 2004; Visser and Bloem 2005; Cham et al. 2007; Prosperini et al. 2014; Surgent et al. 2019). Neurochemical properties of the CNS also influence sway. Visual control mechanisms of balance may be influenced via dopaminergic pathways (Cham et al. 2007).

Nicotine interacts with *dopaminergic structures* in the brain, but also induces balance impairment through cholinergic neurons and nicotinic receptors in the nervous system (Pereira et al. 2001; Staley et al. 2006). Brainstem nicotinic acetylcholine receptors are also involved in the control of breathing (Shao and Feldman 2009). Since H_2_S may act on the respiratory drive through mechanisms that affect brainstem networks (Svendsen 2001), it is relevant to consider cigarette smoking also in studies of motor function in low-dose H_2_S-exposed workers.

In this study of possible nervous system effects among sewage workers exposed to H_2_S primarily in the low-dose area, we differentiate between effects from acute or long-term H_2_S exposure, respectively, and consider a possible modifying effect of cigarette smoking. We emphasize basic motor function, affected also in case studies after acute high exposures, i.e. simple reaction time, balance, or fine motor speed and coordination, rather than higher cognitive function, since we apply internal comparison groups, and the study sample consists of healthy, occupationally active workers with a relatively homogenous educational background.

The specific study aim is to describe a possible association between the H_2_S component of the total exposure, and long-term health effects, emphasizing the motor aspects of neuro-psychological function among wastewater workers.

## Methods

### Study design and setting

This cross-sectional exposure-based study is a part of a larger project, with an overall aim of analysing the complex exposure conditions in the wastewater and sewage industry (Austigard et al. 2018), and outcomes related to pulmonary (Heldal et al. 2019) and nervous system function among workers in such workplaces. Workers in 4 sewage-treatment plants in 2 Norwegian cities (Oslo, Trondheim) and 4 sewage-treatment plants in rural communities or working at the sewer net system with connected sewer pipes and pump stations in the same areas, were invited to participate. The neuropsychological testing took place from February to May 2013. Exposure measurements for the development of a job-exposure H_2_S index took place up to 2015, to cover different work tasks, geographic locations, and seasonal variation.

### Participants, inclusion criteria, and sample size

In total, 148 (99%) of the invited workers accepted to participate; 140 (94.6%) male and 8 (5.4%) female workers. The male workers constituted the study base in the study. Two workers (1.4%) were excluded due to known previous episodes with very high exposure, since they might influence the results strongly in regression analyses, while the most important study objective was possible effects from long-term low-dose H_2_S exposure.

Inclusion was exposure-based. Based on questionnaires of job operations, and workplace inspections, we identified an internal comparison group for acute and sub-acute health effects, that was assessed to be little or not exposed to toxic substances from the sewage at the time of the health examinations. They were workers from a TV-inspection group with minimal contact with wastewater, workers dealing with the fresh-water net system, and administrative personnel with less than 10% exposed working time. They were socioeconomic comparable to the exposed group. Workers relocated to administrative work due to health problems were not included in this group.

The final study base consisted of 138 workers; 41 from sewage treatment plants, 71 from the sewer net system, and 26 low or unexposed referent workers. Most of the latter group had, however, previously been exposed to H_2_S through work. Participant characteristics are described in Table 1. The study was approved by the Regional Medical Ethics Board, decision number 2012/1377. All participants gave their written informed consent.

**Table 1.** Distribution of workplace, age, BMI, smokers, and occupational exposures in the group currently exposed to H_2_S at work (*N* = 112) and the workplace control-group currently unexposed to H_2_S (*N* = 26), and in 4 H_2_S-index subgroups of the total group (*N* = 138).

	Workers currently exposed and unexposed to H_2_S					H_2_S-index—subgroups in the total group								
	Currently exposed *N* = 112		No current exposure *N* = 26			Group 1^a^ *N* = 28 ^b^		Group 2 *N* = 71		Group 3 *N* = 20		Group 4 *N* = 19		
	*N*	%	*N*	%	*P* ^c^	*N*	%	*N*	%	*N*	%	*N*	%	
Sewage plants	41	36.6%	4	15.4%	*-*	28	100.0%	11	15.5%	4	20.0%	2	10.5%	*-*
Sewer net	71	63.4%	22	84.6%	*-*	0	00.0%	60	84.5%	16	80.0%	17	89.5%	*-*
Smokers	25	22.3%	3	11.5%	*0.218*	6	21.4%	15	21.1%	2	10.0%	5	26.3%	*-*
	Mean (SD)^d^	Range	Mean (SD)	Range	*P* ^e^	Mean (SD)	Range	Mean (SD)	Range	Mean (SD)	Range	Mean (SD)	Range	*P* ^e^ ^,^ ^f^
Age, years	45.1 (9.1)	20–63	40.7 (7.8)	26–55	*0.024*	43.0 (11.4)	20–61	44.6 (8.9)	25–63	43.2 (5.7)	34–56	46.0 (8.6)	28–60	*0.336*
BMI	29.0 (5.1)	19.4–52.1	28.7 (5.1)	20.3–43.4	*0.803*	28.2 (5.2)	21.2–43.9	29.3 (4.9)	19.4–52.1	28.5 (4.1)	22.8–38.2	29.2 (6.3)	21.3–8.6	*0.555*
H_2_S-Index	4.3 (2.9)	0.5–10.2	3.8 (2.4)	0.5–9.3	*0.384*	1.0 (0.4)	0.5–1.4	3.3 (0.5)	3.1–4.4	6.7 (0.2)	6.3–7.3	9.7 (0.5)	9.3–10.2	*<0.001*
Endotoxin, EU/m^3^	40.1 (45.0)	0.3–262.0	13.7 (11.3)	0.3 –40.1	*<0.001*	56.4 (57.0)	0.3–240	29.4 (34.7)	0.3–262.0	33.7 (46.6)	0.3–190	26.7 (27.0)	0.3–98	*0.041*
Dust, mg/m^3^	0.31 (0.22)	0.05–1.91	0.33 (0.26)	0.05–1.20	*0.683*	0.32 (0.33)	0.05–1.91	0.32 (0.22)	0.05–1.20	0.33 (0.14)	0.18–0.92	0.31 (0.11)	0.05–0.53	*0.934*

^a^H_2_S-index subgroup values: Group 1 (lowest): 0.54–1.39; Group 2: 3.08–4.40; Group 3: 6.29–7.29; Group 4 (highest): 9.25–10.16.

^b^Distribution of currently H_2_S-exposed versus currently unexposed workers in each subgroup: Group 1: *N* = 24 versus 4; Group 2: *N* = 55 versus 16; Group 3: *N* = 16 versus 4; Group 4: *N* = 17 versus 2.

^c^Pearson chi-square test.

^d^Arithmetic mean (standard deviation).

^e^Independent samples *t*-test, *P*-values in italics.

^f^Group 1 versus Group 4.

### Exposure and comparison groups

To disentangle possible acute effects due to current H_2_S exposure, from the long-term H_2_S component and other exposure components in the work environment, H_2_S exposure was expressed in 2 different variables.

#### Currently exposed and unexposed groups.

A dichotomous exposure variable defined the currently H_2_S-exposed (*N* = 112) and the unexposed referent workers (*N* = 26).

#### Long-term exposure, exposure stratification.

Since both the currently exposed and unexposed workers had been previously exposed to H_2_S through their work, the other variable defined exposure through a JEM for long-term H_2_S exposure (Austigard et al. 2018), considering the severity of exposure, including exposure peaks in various work tasks, besides duration of peaks.

The workers contributed to establishing the exposure level and pattern in the various work situations: During a 1-yr sampling period, to include seasonal variations, airborne endotoxins, inhalable dust particles, and H_2_S were sampled as personal measurements, for approximately 4–5 h from the start of a workday. Each worker registered work operations, use of personal protective device, and breaks during sampling. H_2_S concentrations were measured every 15 s with direct reading instruments. The average of 15 s was recorded.

Twenty-nine percent of the 93 measurements of hydrogen sulphide showed no registered sulphide level. Since active sewage work is likely to involve some generation of H_2_S, and 0.4 was the lowest calculated sulphide index for one measurement, a background sulphide level was estimated at 0.4/√2 = 0.28. All results with 0 sulphide index were replaced with this value. In addition, 37% had exposure ranging up to 1 ppm. Of the remaining 34% with peaks above 1 ppm, 7% points had peaks from 5 to 10 ppm, and 9% of all hydrogen sulphide measurements had exposure peaks above 10 ppm.

A hydrogen sulphide exposure index was estimated based on number of sulphide exposure peaks above 0.1 ppm (H_2_S^01^), 1 ppm (H_2_S^1^), 5.0 ppm (H_2_S^5^) and 10.0 ppm (H_2_S^10^), maximum H_2_S level, and duration of the peaks, to handle the measurements as one value (Austigard et al. 2018):


$$\begin{array}{lll} & H_{2}S\;index=H_{2}S^{01}\times 0.1\;+\;H_{2}S^{Duration01}\times 0.1\;+\; & \;H_{2}S^{1}+\;H_{2}S^{5}\times 5\;+\;H_{2}S^{Duration5}\times 5\;+\;H_{2}S^{10}\times 10\;+\;H_{2}S^{max} \end{array}$$

The index was used in modelling of each participant’s typical workplace exposure. In this neuropsychological study, the participating workers were stratified into 4 exposure sub-groups, where index values in the interval 0.54–1.39 represented the lowest H_2_S exposure (*N* = 28), exposure index values between 3.08 and 4.4 represented the second lowest exposure (*N* = 71), index values between 6.29 and 7.29 represented the second highest (*N* = 20), and between 9.25 and 10.16 the highest long-term exposure to H_2_S (*N* = 19).

A thorough presentation of exposure sampling and the development of the hydrogen sulphide index (Austigard et al. 2018), and a detailed description of work tasks and exposure conditions for the study group, besides results from lung-function examinations, and respiratory and gastro-intestinal symptoms, with the corrected index equation, can be found in previously published studies from the project (Heldal et al. 2019; Austigard and Smedbold 2022).

#### Current smoking.

We compared the neuropsychological outcome among self-reported current smokers (*N* = 28) and non-smokers (*N* = 110) as background information.

### Health examinations and tests

All study participants went through neuropsychological testing, spirometry, and completed self-administrated questionnaires. The questionnaires included questions on smoking and health status, with *gastro-intestinal* and *work-related respiratory* symptoms, and the 18 questions-version of the neurotoxic symptom questionnaire Q16 (Lundberg et al. 1997).

Three neuropsychological tests related to motor function (hand coordination, simple reaction time, and body sway) were included in the test battery. We emphasized tests used in previous sewage worker case studies or patient materials to facilitate comparison. As the participants were generally healthy workers, with similar occupational background, and the purpose was to compare sub-groups according to exposure, cognitive tests for general intellectual function were not included in the test battery as background information.

Grooved Pegboard test from the Halstead-Reitan test battery (Matthews and Kløve 1964) tests hand coordination, i.e. motor speed and fine manipulative dexterity. The test consists of a board with a 5 × 5 set of slotted holes angled in different directions from the centre and 25 identical pegs with a ridge, all of them fitting into each hole. The pegs are to be inserted as quickly as possible into the holes one by one, using only one hand at a time. Completion time for each hand is recorded.

In CATSYS simple auditory reaction time test (SRT) (Després 2000), the subject is holding a handle with a button in the dominant hand and press the button with the thumb as quickly as possible in response to a series of auditory stimuli (beeps) occurring at irregular time intervals. Testing time is 4 min. Average reaction time (msec) and standard deviation are recorded.

The CATSYS postural body-sway test (Danish Product Development 2000) is a Firm Surface Single Platform Force Measure Instrument with sensors mapping the participant’s position from a force centre position (Winter et al. 1996; Prosperini and Pozzilli 2013). The participants stand erect with the feet side-by-side and keep the balance, first with open eyes, thereafter blindfolded. The test duration for each test condition is 75 s, of which the last 60 s are recorded. The test records weight (kg), mean sway, defined as mean of the distance from the geometrical centre of all positions (mm), transversal and sagittal sway, which are the mean of recorded *x* and *y* values of the force centre in a coordinate system with the mean force centre position in the origo (mm), sway area, which is the area of the smallest polygon including all force centre positions (mm^2^), sway intensity, defined by the root mean square of the accelerations recorded in the 0.1–10 Hz band (mm/s^2^), and sway velocity, the average travel period of the force centre calculated by dividing the total length of the force centre trajectory, with the duration (mm/s) (Ellingsen et al. 2008, 2015).

Similar tests have been used in previous studies of H_2_S toxicity, but in symptom rather than exposure-based study samples (Kilburn 1997, 2003; Guidotti 2010).

The order of the neuropsychological tests was the same for each participant, and all were tested by the same examiner. Each test session took place one of the days Tuesday to Thursday during the daytime between 10 a.m. and 2 p.m., and completion time was approximately 30 min.

### Statistical analyses

Background and outcome variables, and exposure, were summarized with mean, standard deviation (SD), minimum, and maximum values. *Acute or subacute effects* were studied by comparing the currently H_2_S-exposed with the currently unexposed workers, using Pearsons chi-square test for the categorical questionnaire outcome variables, and independent samples *t*-test for the continuous neuropsychological test variables. To assess *long-term health effects*, the 4 stratified H_2_S exposure-index groups, comparing the lowest exposed with higher exposed workers, were used to achieve a possible dose–response profile, also using Pearsons chi-square test for categorical, and independent samples *t*-test for the continuous outcome variables. We applied independent samples *t*-test when comparing test performance among smokers and non-smokers. Spearman’s nonparametric correlation was used to assess the correlation between selected independent exposure or background variables considered for inclusion into a regression model. We used multiple linear regression to assess associations between the independent variables age, and H_2_S-exposure variables, and dependent continuous outcome variables. The regression analyses were done in the total study sample, and in each of the sub-groups of smokers and non-smokers, to identify possible differential outcome patterns. The outcome variables were each neuropsychological test parameter. Test raw scores were applied, and age was included, because sensory-motor neuropsychological test performance is known to be age-dependent. All analyses were performed using IBM SPSS Statistics 20 (IBM, Armonk NY).

## Results

In the total study sample (*N* = 138), *N* = 45 (32.6%) worked in sewage plants, and *N* = 93 (67.4%) in the sewer net. The distribution of background and exposure variables was as follows: Mean age (SD) was 44.3 (9.0) years, range 20–63, mean employment duration was 10.0 (9.36) years, range 0.1–39, with a significant correlation between age and employment duration (*r* = 0.41, *P* < 0.001). Body mass index (BMI) was 29.0 (5.0) kg/m², range 19.4–52.1, and *N* = 28 (20.3%) were current smokers. The mean H_2_S-index level (SD) was 4.2 (2.8), range 0.5–10.2. The mean endotoxin exposure level was 35.1 (42.1) EU/m^3^, range 0.3–262.0. The mean dust exposure level was 0.32 (0.23) mg/m^3^, range 0.05–1.91.

The distribution of background and exposure variables in the H_2_S-exposed and referent groups, and subgroups stratified according to the H_2_S index, are shown in Table 1. The Table 1 exposure stratification indicates that H_2_S exposure was higher in the sewer net than in sewage plants. Heldal et al. (2019) further specifies that higher H_2_S exposure is more common in *rural* plants and net system, particularly when collecting sewage from cesspools.

There was a slight statistical nonsignificant negative correlation (Spearman nonparametric test) between the H_2_S index and endotoxin exposure (*r* = −0.13, *P* = 0.128), and a statistical significant correlation between the H_2_S index and dust exposure (*r* = 0.28, *P* < 0.001) in the total study sample (*N* = 138).

Smokers and non-smokers had similar mean H_2_S exposure-index values. The proportion of smokers and non-smokers currently exposed to H_2_S at work were also comparable. The smokers were 2.6 years older than the non-smokers (Table 4). There were no significant differences between smokers and non-smokers in exposure to dust (0.28 versus 0.33 mg/m^3^, *P* = 0.390, independent samples *t*-test) or endotoxins (43.1 versus 33.1 EU/m^3^, *P* = 0.263), despite higher level of particularly endotoxins among smokers.

**Table 4.** Distribution of age, BMI, occupational exposures to H_2_S at work, postural sway, and neurobehavioral motor tests Grooved Pegboard and Simple Reaction Time (SRT) among current smokers (*N* = 28) and non-smokers (*N* = 110).

	Non-smokers (*N* = 110)	Smokers (*N* = 28)	*P* ^a^
	Mean (SD)^b^	Mean (SD)^b^	
Age, years	43.7 (9.1)	46.3 (8.6)	*0.181*
BMI, kg/m²	28.9 (4.9)	29.3 (5.7)	*0.803*
Current H_2_S exposure (*N*, %)	87 (79.1)	25 (89.3)	*-*
H_2_S-Index	4.2 (2.7)	4.2 (3.0)	*0.962*
Postural sway Eyes open			
Transversal x (mm)	2.8 (0.9)	2.8 (0.8)	*0.784*
Sagittal *y* (mm)	4.2 (1.4)	4.6 (2.0)	*0.166*
Sway area (mm^2^)	244 (104)	286 (106)	*0.060*
Mean sway (mm)	5.5 (1.6)	6.0 (2.1)	*0.213*
Intensity (mm/s^2^)	3.9 (0.9)	4.2 (0.8)	*0.101*
Sway velocity (mm/s)	**9.8 (2.5)**	**11.0 (3.0)**	***0.036***
*Blindfolded*			
Transversal x (mm)	4.0 (1.4)	4.1 (1.2)	*0.756*
Sagittal *y* (mm)	4.9 (1.4)	5.3 (1.5)	*0.140*
Sway area (mm^2^)	584 (335)	670 (274)	*0.209*
Mean sway (mm)	7.0 (1.9)	7.5 (1.9)	*0.289*
Intensity (mm/s^2^)	6.3 (1.7)	7.0 (1.5)	*0.053*
Sway velocity (mm/s)	**18.6 (6.8)**	**21.6 (7.4)**	***0.045***
*Grooved Pegboard*			
Right hand (sec)	**63.6 (11.4)**	**70.1 (9.1)**	***0.006***
Left hand (sec)	**67.9 (11.7)**	**73.3 (12.0)**	***0.033***
*Simple Reaction Time*			
Mean (msec)	221.6 (27.8)	228.2 (36.5)	*0.295*
SD (msec)	46.9 (20.0)	43.3 (13.6)	*0.261*

^a^Independent samples *t*-test, *P*-values in italics,bold values; *P* < 0.05.

^b^Arithmetic mean (standard deviation).

### Work-related symptoms

In the total study sample, symptoms from the nose (*N* = 82, 59.9%), coughing (*N* = 75, 54.7%), nausea (*N* = 58, 42.0%), wheezing (*N* = 54, 39.4%), and fever attack (*N* = 50, 36.2%), were the most frequently reported respiratory symptoms. Heavy breathing and airway symptoms were reported by *N* = 41 (30.1%) and *N* = 40 (29.2%), respectively. Of the CNS symptoms, tiredness (*N* = 63, 46.3%), forgetfulness (*N* = 63, 46.3%), and concentration difficulties (*N* = 43, 31.6%), were most often reported. Also, while no participants without current exposure to H_2_S reported palpitations, *N* = 10 (9.1%) of the currently H_2_S-exposed did. Currently, H_2_S-exposed workers at sewage plants and sewage net system showed no significant difference in symptom reporting.

The prevalence of *current work-related respiratory symptoms* was nonsignificantly elevated among workers compared to referents regarding heavy breathing, and significantly higher for airway symptoms (Supplementary Table A), while these two symptoms were equally prevalent among non-smokers as among smokers (*N* = 33, 30.3% versus *N* = 8, 29.6%, *P* = 1.000, and *N* = 32, 29.4% versus *N* = 8, 28.6%, *P* = 1.000, respectively) in our study sample.

No systematic dose–response relationship between sub-groups stratified according to the H_2_S exposure index was observed. Acute coughing only, tended to be reported significantly more often in the sub-group with the highest, compared to the lowest, estimated H_2_S exposure.

### Neuropsychological data

Total study sample mean body-sway area was 253 mm^2^ (SD = 106) with open eyes, and 602 mm^2^ (SD = 325) when blindfolded. Body sway was not significantly different in the currently H_2_S-exposed workplace group (*N* = 112) compared with the unexposed control group (*N* = 26). When stratified into 4 exposure sub-groups according to the H_2_S exposure-index level, we observe a tendency of nonsignificantly higher postural body-sway area when blindfolded, with increasing H_2_S index, from 581 mm^2^ in the 2 lowest H_2_S-index sub-group values, to 702 mm^2^ in the sub-group with the highest H_2_S-index value (Table 2).

**Table 2.** Postural Sway and neurobehavioral motor tests Grooved Pegboard and Simple Reaction Time (SRT) for currently exposed male sewage workers (*N* = 112) and workplace control-group/internal referents (*N* = 26) of currently unexposed workers, and in H_2_S-index sub-groups of the total group (*N* = 138).

	Workers currently exposed and unexposed to H_2_S					H_2_S-index—subgroups in the total group								
	Currently exposed *N* = 112		No current exposure *N* = 26			Group 1^a^ *N* = 28 ^b^		Group 2 *N* = 71		Group 3 *N* = 20		Group 4 *N* = 19		
	Mean (SD)^c^	Range	Mean (SD)	Range	*P* ^d^	Mean (SD)	Range	Mean (SD)	Range	Mean (SD)	Range	Mean (SD)	Range	*P* ^d^ ^,^ ^e^
*Postural sway* *Eyes open*														
Transversal x (mm)	2.8 (0.8)	1.6–5.0	2.8 (1.3)	1.4–7.8	*0.916*	2.6 (0.6)	1.7–4.5	2.9 (1.0)	1.4–7.8	2.6 (0.8)	1.5–4.6	2.8 (0.8)	1.6–4.6	*0.558*
Sagittal *y* (mm)	4.3 (1.6)	1.9–12.5	4.1 (1.5)	2.0–8.3	*0.561*	4.2 (1.5)	2.3–9.4	4.4 (1.8)	1.9–12.5	4.0 (1.4)	2.0–8.3	3.9 (1.0)	2.0–6.3	*0.486*
Sway area (mm^2^)	257 (107)	92–644	232 (101)	78–537	*0.282*	245 (89)	92–458	260 (108)	78–537	235 (129)	92–644	252 (100)	98–462	*0.801*
Mean sway (mm)	5.6 (1.7)	2.9–13.2	5.5 (1.7)	2.8–9.1	*0.742*	5.5 (1.5)	3.2–9.7	5.8 (1.8)	2.8–13.2	5.3 (1.6)	2.9–9.1	5.3 (1.3)	3.2–7.6	*0.691*
Intensity (mm/s^2^)	3.9 (0.8)	2.3–6.2	3.6 (0.9)	2.3–5.4	*0.040*	3.7 (0.7)	2.3–4.9	4.0 (0.9)	2.4–5.9	3.9 (1.0)	2.3–6.2	4.0 (0.8)	2.8–5.9	*0.303*
Sway velocity (mm/s)	10.2 (2.6)	5.4–18.5	9.4 (2.4)	6.7–14.7	*0.193*	10.3 (2.9)	6.2–18.5	9.9 (2.3)	6.1–16.9	9.7 (3.3)	5.4–17.8	10.5 (2.5)	7.0–17.7	*0.816*
*Blindfolded*														
Transversal x (mm)	4.1 (1.4)	1.3–9.8	3.9 (1.0)	2.2–6.7	*0.435*	3.8 (1.1)	2.2–6.9	4.0 (1.3)	1.3–8.9	4.1 (1.4)	1.9–7.2	4.5 (1.9)	2.3–9.8	*0.111*
Sagittal *y* (mm)	5.0 (1.5)	2.6–10.2	4.9 (1.1)	2.6–8.0	*0.677*	4.8 (1.4)	2.6–8.4	4.9 (1.3)	2.6–9.5	5.1 (1.4)	3.3–7.9	5.3 (1.8)	3.0–10.2	*0.296*
Sway area (mm^2^)	616 (338)	111–1918	540 (258)	200–1377	*0.280*	581 (352)	263–1786	581 (278)	111–1377	610 (360)	223–1455	702 (410)	231–1918	*0.286*
Mean sway (mm)	7.2 (2.0)	3.8–14.4	6.9 (1.4)	4.4–10.0	*0.415*	6.8 (1.8)	3.9–11.9	7.1 (1.8)	3.8–12.7	7.3 (1.9)	4.2–10.8	7.7 (2.6)	4.7–14.4	*0.151*
Intensity (mm/s^2^)	6.5 (1.7)	3.5–12.2	6.2 (1.6)	3.7–11.6	*0.356*	6.5 (1.7)	3.6–10.3	6.4 (1.5)	3.5–11.6	6.2 (2.1)	3.5–12.1	6.9 (2.0)	4.2–12.2	*0.480*
Sway velocity (mm/s)	19.5 (7.0)	8.4–44.2	17.7 (6.9)	9.6–38.2	*0.232*	19.4 (7.6)	10.9–38.8	18.7 (6.2)	8.4–38.2	19.5 (9.9)	8.8–44.2	20.6 (5.09)	10.5–32.7	*0.559*
*Grooved Pegboard (sec)*														
Right hand	64.7 (11.1)	48–120	65.8 (11.9)	51–99	*0.652*	62.2 (8.6)	50–80	66.0 (12.5)	49–120	63.8 (9.7)	51–81	66.1 (11.5)	48–90	*0.187*
Left hand	69.5 (12.5)	48–116	67.1 (9.0)	50–87	*0.358*	67.2 (8.8)	51–88	69.4 (11.5)	51–103	65.9 (10.2)	50–84	73.6 (17.4)	48–116	*0.103*
*Simple reaction time*														
SRT mean (msec)	**225.8 (29.9)**	**171**–**347**	**210.7 (26.3)**	**160**–**255**	***0.019***	226.1 (28.5)	189–284	223.3 (29.6)	171–347	223.9 (28.0)	172–276	215.6 (34.4)	160–282	*0.259*
SD (within test, msec)	**48.2 (19.3)**	**16**–**109**	**37.5 (14.1)**	**21**–**70**	***0.009***	48.9 (21.8)	22–109	47.4 (18.4)	16–97	38.7 (14.6)	16–69	45.3 (19.3)	23–84	*0.567*

^a^H_2_S-index subgroup values: Group 1 (lowest): 0.54–1.39; Group 2: 3.08–4.40; Group 3: 6.29–7.29; Group 4 (highest): 9.25–10.16.

^b^Distribution of currently H_2_S-exposed versus currently unexposed workers in each subgroup: Group 1: *N* = 24 versus 4; Group 2: *N* = 55 versus 16; Group 3: *N* = 16 versus 4; Group 4: *N* = 17 versus 2.

^c^Arithmetic mean (standard deviation).

^d^Independent samples *t*-test, *P*-values in italics, bold values; *P* < 0.05.

^e^Group 1 versus Group 4, No significant differences when comparing the two highest with the two lowest exposure subgroups.

Smokers tended to have nonsignificantly larger body-sway *area* than non-smokers, while body-sway *velocity* was significantly larger among smokers, both when tested with the eyes open and closed (Table 4).

The multivariate regression analyses did not reveal consistent statistical associations between the independent variables and body sway in the total study sample, neither with the eyes open during testing, nor blindfolded, when including age (also a proxy for employment duration) and the 2 H_2_S-exposure variables in the analyses (Table 3). When repeating the regression analysis among smokers and non-smokers separately (Table 5), the analyses indicate a significant contribution among smokers only, of the H_2_S index, in explaining the body-sway test results in the blindfolded condition. The explained model variance (*R*^2^), although statistical nonsignificant, is also higher among the smokers than in the non-smoking sub-group, in the blindfolded testing condition. The results indicate a differential pattern of associations between the H_2_S index and the blindfolded body-sway test, in smokers and non-smokers.

**Table 3.** Postural sway with eyes open and blindfolded, Grooved Pegboard and Simple reaction Time (SRT) as a function of age and occupational exposure in male sewage workers, total group (*N* = 138): Linear regression^a^.

Raw scores	Constant		Regression coefficients^b^						*R* ^2^	Model *P*-value
			Age		H_2_S index^c^		Current H_2_S			
	*B*	*P*-value	*B*	*P*-value	*B*	*P*-value	*B*	*P*-value		
*Postural sway* *Eyes open*										
Transversal x (mm)	2.4	*<0.001*	0.01	*0.236*	−0.007	*0.810*	−0.06	*0.753*	0.01	*0.691*
Sagittal *y* (mm)	4.6	*<0.001*	−0.01	*0.686*	−0.06	*0.258*	0.26	*0.466*	0.01	*0.608*
Sway area (mm^2^)	148.2	*0.002*	2.21	*0.031*	−1.46	*0.652*	15.8	*0.497*	0.04	*0.113*
Mean sway (mm)	5.7	*<0.001*	0.001	*0.969*	−0.055	*0.293*	0.15	*0.695*	0.01	*0.748*
Intensity (mm/s^2^)	3.1	*<0.001*	0.01	*0.190*	0.013	*0.627*	0.33	*0.084*	0.05	*0.101*
Sway velocity (mm/s)	8.3	*<0.001*	0.03	*0.275*	−0.003	*0.967*	0.62	*0.287*	0.02	*0.410*
*Blindfolded*										
Transversal x (mm)	3.6	*<0.001*	−0.001	*0.959*	0.07	*0.086*	0.20	*0.515*	0.03	*0.312*
Sagittal *y* (mm)	4.5	*<0.001*	0.004	*0.761*	0.06	*0.193*	0.08	*0.800*	0.02	*0.570*
Sway area (mm^2^)	469.2	*0.002*	0.50	*0.873*	13.2	*0.190*	67.5	*0.353*	0.02	*0.401*
Mean sway (mm)	6.4	*<0.001*	0.004	*0.844*	0.10	*0.091*	0.20	*0.636*	0.03	*0.337*
Intensity (mm/s^2^)	5.4	*<0.001*	0.02	*0.326*	0.02	*0.663*	0.26	*0.496*	0.02	*0.561*
Sway velocity (mm/s)	14.3	*<0.001*	0.07	*0.285*	0.13	*0.561*	1.45	*0.356*	0.02	*0.396*
*Grooved Pegboard*										
Right hand (sec)	45.8	*<0.001*	**0.49**	***<0.001***	0.04	*0.893*	−3.29	*0.161*	**0.15**	***<0.001***
Left hand (sec)	41.1	*<0.001*	**0.62**	***<0.001***	0.19	*0.570*	−0.46	*0.844*	**0.22**	***<0.001***
*Simple reaction time*										
SRT mean (msec)	205.2	*<0.001*	0.24	*0.390*	−1.18	*0.192*	**14.7**	***0.026***	**0.06**	***0.050***
SD (within test, msec)	29.0	*<0.001*	0.30	*0.097*	−0.93	*0.100*	**9.92**	***0.016***	**0.09**	***0.007***

^a^Linear regression. Dependent variable: Each sway, pegboard, or SRT-variable. Independent variables: Age (years), H_2_S exposure (index), H_2_S exposure (current, yes/no), included in total-group model.

^b^Unstandardized B-coefficient shown for all variables included in the model, *P*-values in italics, bold values; *P* < 0.05.

^c^H_2_S-index, exposure-level individual raw scores applied, rather than H_2_S-index subgroup values.

**Table 5.** Postural sway with eyes open and blindfolded, Grooved Pegboard and Simple reaction Time (SRT) as a function of age and occupational exposure in male sewage workers, total group (*N* = 138), and stratified according to smoking status: Linear regression^a^.

Raw scores	Constant		Non-smokers (*N* = 110)						*R* ^2^	Model *P*-value	Constant		Current smokers (*N* = 28)						*R* ^2^	Model *P*-value
			Regression coefficients ^b^										Regression coefficients^b^							
			Age		H_2_S index^c^		Current H_2_S						Age		H_2_S index^c^		Current H_2_S			
	*B*	*P*-value	*B*	*P*-value	*B*	*P*-value	*B*	*P*-value			*B*	*P*-value	*B*	*P*-value	*B*	*P*-value	*B*	*P*-value		
*Postural sway*																				
*Eyes open*																				
Transversal x (mm)	2.5	*<0.001*	0.01	*0.392*	−0.03	*0.348*	0.1	*0.784*	0.02	*0.664*	2.0	*0.043*	0.02	*0.203*	0.06	*0.294*	−0.6	*0.275*	0.16	*0.236*
Sagittal *y* (mm)	4.0	*<0.001*	0.01	*0.568*	−0.09	*0.069*	0.2	*0.549*	0.03	*0.299*	7.4	*0.008*	−0.08	*0.107*	0.05	*0.750*	0.7	*0.596*	0.11	*0.400*
Sway area (mm^2^)	149.0	*0.004*	2.16	*0.054*	−3.96	*0.281*	21.7	*0.384*	0.05	*0.127*	175.1	*0.222*	2.08	*0.416*	5.57	*0.471*	−10.0	*0.891*	0.05	*0.755*
Mean sway (mm)	5.3	*<0.001*	0.01	*0.439*	−0.10	*0.060*	0.2	*0.643*	0.04	*0.258*	7.9	*0.007*	−0.06	*0.263*	0.08	*0.588*	0.3	*0.819*	0.07	*0.627*
Intensity (mm/s^2^)	2.9	*<0.001*	0.02	*0.118*	0.002	*0.952*	0.4	*0.073*	0.06	*0.071*	4.7	*<0.001*	−0.01	*0.580*	0.02	*0.776*	−0.1	*0.847*	0.03	*0.895*
Sway velocity (mm/s)	8.4	*<0.001*	0.02	*0.446*	0.03	*0.743*	0.6	*0.361*	0.02	*0.563*	10.4	*0.014*	0.03	*0.702*	−0.13	*0.546*	−0.2	*0.913*	0.03	*0.894*
*Blindfolded*																				
Transversal x (mm)	3.9	*<0.001*	−0.01	*0.627*	0.04	*0.397*	0.3	*0.355*	0.02	*0.594*	1.8	*0.224*	0.03	*0.204*	**0.18**	***0.036***	−0.1	*0.953*	**0.22**	*0.113*
Sagittal *y* (mm)	4.4	*<0.001*	0.01	*0.555*	0.01	*0.859*	0.1	*0.871*	0.01	*0.919*	4.4	*0.025*	−0.02	*0.625*	**0.24**	***0.026***	0.8	*0.401*	**0.21**	*0.132*
Sway area (mm^2^)	568.9	*<0.001*	−1.70	*0.643*	8.3	*0.488*	68.7	*0.401*	0.01	*0.695*	−67.2	*0.838*	9.82	*0.106*	**38.70**	***0.039***	134.2	*0.433*	**0.23**	*0.097*
Mean sway (mm)	6.5	*<0.001*	0.003	*0.895*	0.04	*0.556*	0.3	*0.583*	0.01	*0.835*	5.0	*0.037*	0.01	*0.773*	**0.32**	***0.015***	0.7	*0.582*	**0.23**	*0.100*
Intensity (mm/s^2^)	5.8	*<0.001*	0.01	*0.739*	0.01	*0.855*	0.3	*0.494*	0.01	*0.850*	4.6	*0.022*	0.05	*0.159*	0.07	*0.490*	−0.1	*0.914*	0.09	*0.497*
Sway velocity (mm/s)	16.1	*<0.001*	0.02	*0.834*	0.22	*0.373*	1.2	*0.486*	0.02	*0.636*	9.5	*0.328*	0.25	*0.160*	−0.05	*0.925*	−0.7	*0.883*	0.09	*0.516*
*Grooved Pegboard*																				
Right hand (sec)	42.4	*<0.001*	**0.56**	***<0.001***	−0.01	*0.988*	−4.0	*0.114*	**0.19**	***<0.001***	71.2	*<0.001*	0.04	*0.868*	−0.02	*0.982*	−3.1	*0.629*	0.01	*0.965*
Left hand (sec)	42.4	*<0.001*	**0.60**	***<0.001***	−0.06	*0.865*	−1.0	*0.696*	**0.21**	***<0.001***	35.2	*0.018*	0.68	*0.012*	0.84	*0.279*	3.5	*0.635*	0.26	*0.062*
*Simple reaction time*																				
SRT mean (msec)	204.1	*<0.001*	0.29	*0.326*	−0.81	*0.409*	10.2	*0.127*	0.04	*0.247*	207.9	*<0.001*	−0.15	*0.854*	−1.46	*0.563*	37.5	*0.125*	0.14	*0.291*
SD (within test, msec)	25.7	*0.007*	0.41	*0.052*	−1.25	*0.069*	**11.1**	***0.018***	**0.11**	***0.006***	35.9	*0.054*	−0.01	*0.979*	−0.21	*0.826*	9.7	*0.299*	0.06	*0.677*

^a^Linear regression. Dependent variable: Each sway, pegboard, or SRT variable. Independent variables: Age (years), H_2_S exposure (index), H_2_S exposure (current, yes/no), included in model for smokers or no-smokers, respectively.

^b^Unstandardized *B* coefficient shown for all variables included in the model, *P*-values in italics, bold values; *P* < 0.05.

^c^H_2_S-index, individual exposure-level raw-scores applied, rather than H_2_S-index subgroup values.

Fine motor speed/eye-hand coordination in the total study sample, as measured with the Grooved Pegboard test, was 64.9 s (SD = 11.3) in the right hand, and 69.0 s (SD = 11.9) in the left hand, with no significant differences between currently H_2_S-exposed and unexposed, and with no tendencies across the exposure-index level (Table 2). However, smokers needed significantly longer time than non-smokers to complete this test in both hands (Table 4). The multivariate regression analyses indicate an association between increasing age and prolonged completion time in both hands, in both the total study sample (Table 3) and the sub-samples of smokers and non-smokers, respectively (Table 5).

The mean SRT was 222.9 ms (SD = 29.7), and the within-person reaction time SD was 46.2 ms (SD = 18.9) in the total study sample (*N* = 138). The SRT was significantly longer, and the within-person SRT dispersion significantly larger, in the currently exposed workplace group (*N* = 112) compared with the faster-unexposed control group (*N* = 26) (Table 2), while SRT among smokers was not significantly longer than non-smokers (Table 4). In the total study sample (Table 3), but not in the sub-groups based on smoking status (Table 5), the regression analyses also indicate a significant association between current H_2_S exposure and longer SRT.

## Discussion

The analyses indicate an association between current sewage work, and increased SRT, in the total study sample. *Body-sway tested blindfolded* indicates a statistically significant dose-dependent negative impact on balance *among smokers,* but not among *non-smokers*, of typical workplace long-term H_2_S exposure, as reflected by the H_2_S index. Balance is more impaired in the highest compared to the less H_2_S-exposed subgroups of our total study sample, but our lowest H_2_S-exposed sub-groups are *not improved* compared to controls, thus not indicating any stimulating effect from low-level H_2_S exposure. *Body-sway tested with open eyes* is in the normal range. We observe no statistically significant negative impact of neither the ongoing nor long-term H_2_S exposure, on work-related nervous system symptoms, or fine-motor coordination.

Our analyses show slightly higher symptom prevalence among currently exposed workers compared to referents, but very few statistically significant differences. The level of symptom reporting is comparable to previous studies (Melbostad et al. 1994,; Douwes et al. 2001; Lee et al. 2007). *Reduced concentration, memory problems*, and *being tired* are frequently reported among sewage workers (Lewis and Copley 2015), as is also the case in our study. Since the controls are previously exposed, but *currently* hardly exposed, to toxicants from the sewage, the limited group difference trends may support an assumption of limited H_2_S exposure with consecutive limited *acute reactions* in the exposed group. Alternatively, exposure misclassification regarding current exposure to H_2_S and other toxins, due to, for instance, recreational farming or other unknown occupational exposure particularly in the currently unexposed group, could also cause reduced group differences. This, however, we consider less probable, since both groups are employees in the same companies, with full-time work, and with limited opportunities for alternative exposure-related activities.

If this is correct, the analyses represent a check of whether acute effects from current H_2_S exposure could conceal health effects from possible long-term exposure in analyses based on the H_2_S index. The analyses did not indicate this, and thus justified consideration of trends in CNS symptoms or neuropsychological test performance based on the exposure index.

We observed no systematic dose-response pattern of increasing self-reported symptom levels or reduced test performance, with higher H_2_S-index level, that could indicate long-term effects primarily from H_2_S alone. Since the exposed versus control group differences were also limited, the slightly elevated symptom prevalence in the exposed group may as likely be due to other aspects of the total exposure in sewage plants, like dust, endotoxins, or bacteria (Melbostad et al. 1994; Douwes et al. 2001), not further considered in this study. Heavy breathing or airway symptoms might reflect high physical workload, that may affect test performance through increased respiration and subsequent biological exposure to H_2_S. However, as these symptoms are reported by less than a third of the participants, and are equally prevalent among non-smokers and smokers, this most likely does not explain such outcome among smokers only.

Significantly longer SRT in the currently H_2_S-exposed group, compared to unexposed controls, however, was also observed by Farahat and Kishk (2010). Since SRT is comparable in the lowest and the highest exposed H_2_S exposure-index sub-group (Table 2), and there are no significant associations between SRT and the H_2_S-index in the multivariate analyses (Table 3), the prolonged SRT in the currently exposed group may be interpreted primarily as a reaction to the acute/sub-acute H_2_S exposure.

The overall normal test results may be due to the general low exposure level in our study sample, or that H_2_S does not strongly affect our chosen outcome variables but may also be due to methodological issues. We had to rely on *self-reported smoking* in our study, as we had not included biomarkers of nicotine. Misclassification regarding smoking status would tend to reduce group differences in effects, as the snuffers would appear as non-smokers, although they were nicotine exposed. However, in our smokers, sway velocity is also significantly increased, also reported by Pereira et al. (2001), and Grooved Pegboard is significantly slower, also observed by Nadar et al. (2021) (Table 4). Such behavioural response pattern supports that the self-reported allocation in smoking status in our study may be sufficiently valid.

The observed dose-dependent association between the sway area *tested blindfolded* and the H_2_S index among smokers only (Table 5) may indicate that the H_2_S exposure alone is not sufficient for developing health effects. That we see this in otherwise healthy workers, at lower exposure levels than is previously assumed to lead to health effects, represents a new observation.

Mechanisms regarding nervous system contributions to balance deficits are not fully understood (Welniarz et al. 2021), and careful mechanistic inferences only, should be drawn from our primarily neurobehavioral observations.

When feet are placed close, parallel and side-by-side, anterior-posterior (A/P) sway is under *ankle-control*, while medial-lateral (M/L) balance is under *hip control* (Winter et al. 1996). Sway area combines these measures.

Sway may be influenced by various chemical agents; occupational after severe H_2_S intoxications through anoxic or direct mechanisms (Kilburn and Warshaw 1995, 1997, 2003), or after manganese exposure in welders (Ellingsen et al. 2008, 2015), or life style, like cigarette smoking or alcohol consumption (Pereira et al. 2001; Staley et al. 2006). Degenerative nervous system diseases like MS or Parkinson’s disease (Prosperini et al. 2014) also impair balance. Balance is gradually impaired in the elderly, and falling due to impaired balance may have severe health consequences and high costs (Kinge et al. 2023).

Since we observed a dose-dependent increased sway area when tested with closed eyes only, while sway tested with open eyes was normal, and the sway area in our study group had no specific direction in the A/P direction, this disregards a role of both dopaminergic pathways that are also associated with A/P sway (Cham et al. 2007), and certain parts of the cerebellum (Morton and Bastian 2004), also associated with A/P sway.

The exposure–effect profile in our study does not indicate that it is immediately biologically plausible to assume that endotoxins or dust exposure should affect balance/SWAY negatively.

Since we observed an association between H_2_S exposure and balance in smokers only, one could speculate that nicotine and H_2_S act on similar neuronal structures in the CNS, and that cholinergic receptors or nerves in the brainstem or basal ganglia may be a possible area of toxic effects from H_2_S (Pereira et al. 2001; Staley et al. 2006; Shao and Feldman 2009). But, *further studies are warranted* to gain increased understanding of these possible exposure—effect associations.

Neither the currently exposed group nor the sub-group with the lowest H_2_S-index values did systematically better than the currently unexposed controls on the neuropsychological tests. Our study accordingly does not support any stimulating effects of low-dose H_2_S exposure at levels present in the sewage work in our study, compatible with H_2_S as an endogenously produced nervous system gasotransmitter (Abe and Kimura 1996; Kimura 2011; Zhang et al. 2017) that was observed in population studies of low dose ambient H_2_S exposure (Inserra et al. 2004; Reed et al. 2014), or in experimental chamber studies of low-dose H_2_S exposure (Fiedler *et al.*, 2008).

However, both level and pattern of exposure may be different in the community studies compared to our occupational study; Exposure levels above 1 ppm are very uncommon in community settings (Guidotti 2010), while the general low exposure level in the work environment, with 8-h time-weighted average (TWA) measurements below 5 ppm, is accompanied by occasional more frequent and higher situational or task-related exposure peaks above 10 ppm, and all groups with active sewage work may even have incidents of exposure above 100 ppm (Austigard et al. 2018, 2023; Austigard and Smedbold 2022).

The impact of the occasional peak pattern for the observed outcomes related to sway or balance is supported, since reduced performance in neuropsychological examination was not demonstrated in the community studies, after pure low-dose H_2_S exposure in the ppb range and below 5 ppm, without exposure peaks. People exposed to ambient low-dose H_2_S rather performed slightly better on tests of basic motor functions.

This implies that concerns about pure low-dose exposure to H_2_S may be reduced but also that concerns regarding repeated exposure peaks in the work atmosphere should be maintained (Austigard et al. 2023).

### Study design, study sample, methods

The H_2_S sampling strategy included measurements for 1 yr, and the H_2_S-exposure index emphasized each worker’s various work operations at the workplace. Strictly speaking, the index is based on and primarily represents exposure sampling during the year close in time to the health examination only. The intention was, however, to make an index that reflected an overall *typical* exposure for this kind of work, also beyond the actual sampling period, so that it was justified to label this *long-term typical*, although not cumulative, H_2_S exposure (Austigard et al. 2018), in contrast to acute or subacute short-term H_2_S exposure, that may vary independent of the typical exposure, during the day or few days preceding the health examinations. Such approach could also be justified, since H_2_S is not accumulated in the body, but is easily transformed and excreted in urine (Farahat and Kishk 2010).

The approach presupposes that high-exposure situations and patterns during the year of exposure sampling may, to some extent, resemble the exposure pattern of the previous years. Still, we cannot rule out that *the typical general exposure may have decreased the more recent years*. Such possible underestimation of typical long-term exposure prior to the year of exposure sampling could lead to an overestimation of health effects from H_2_S, based on the index, which would also be the case if the index did not sufficiently reflect the high-dose peak exposures.

Such challenges have been discussed by several authors. Long-term exposure indexes are most often composed of both duration and amount of exposure. However, different authors have assumed that either all previous exposure are weighted equally, or that *more recent exposure*, or the last 5 yr, should be given extra weight (Ruijten et al. 1990). Higher *general levels of exposure* have also been given extra weight compared to lower levels (De Fruyt et al. 1998). The index in our study, in addition, gives *extra weight to peak exposures* (Austigard et al. 2018). Thus, there is no consensus on how to construct an exposure index.

Another challenge is that although each worker is assigned an individual index value, the exposure measurements used in construction of the index are associated with the typical exposure at the workplace and -tasks. All workers with similar “work-activity profile” will thus be assigned the same index value, although the individual exposure may vary between them, making the index a cruder exposure indication when studying exposure—effect associations.

Neuropsychological test results in previous epidemiological studies have been difficult to interpret in terms of CNS-dysfunction alone, due to inclusion based on manifest self-reported symptoms (Kilburn et al. 2010), tending to exaggerate health effects from low-dose H_2_S exposure. The *exposure-based inclusion* into our study, and the close to complete *participation rate*, minimizes the risk of selective inclusion into the study and reduces the likelihood of a symptom-based bias in the study sample. A study sample of occupationally active workers, however, increases the likelihood of minimizing health effects, due to a healthy-worker effect. Exposure misclassification could in addition make us overlook real differences in acute health outcome between the currently exposed and unexposed groups. However, as already described, we consider a bias due to pronounced exposure misclassification regarding current exposure, less likely.

The exposure-index stratification approach for long-term effects, based on measurements rather than reporting, may reduce a possible symptom bias from differential worry among employees regarding effects from low-dose exposures, as such worry may be distributed incidentally among the H_2_S-index sub-groups, not contributing systematically to possible observed dose-effect trends.

Our dual approach to the *study design* may thus have enabled analysis of health effects from both short-term acute or subacute-, and long-term typical H_2_S exposure, separately. We first checked in the cross-sectional exposed versus control approach. Since the most recent H_2_S-exposure was apparently not important for test performance at the H_2_S-exposure levels in our study, a study of the outcome pattern at 4 different long-term index levels in the exposure-index stratification approach (Table 2 and Supplementary Appendix A) and use of linear regression analyses (Tables 3 and 5), to disentangle whether long-term H_2_S exposure contributed to the results, was possible.

It must be added, though, that the approach is dose-related: When the ongoing typical exposure is generally low or limited, we may expect minor *acute effects* in the currently H_2_S-exposed group and minor (nonsignificant) differences between the currently exposed versus control group. But with more pronounced ongoing work-related exposure in our study sample, we would expect to observe significant effect differences between the currently exposed and unexposed employees and would not be able to study long-term effects separately.

Our approach presupposes that exposure group allocation is sufficiently valid, and not subject to control group exposure misclassification, and that the current level of exposure to H_2_S is generally low.

The neuropsychological tests, with a focus on motor function and coordination, were chosen because they could be compared to results from other occupational, community, or experimental studies of H_2_S exposure. Since the test choice was based on a priori hypotheses, with variables that were not independent of each other, we did not correct for the magnitude of statistical analyses. If we had not based our study on biological plausible hypotheses, we would be more vulnerable to incidental associations or exaggeration of findings. Contrary to this, some nonsignificant tendencies might have been statistically more pronounced, if our study sample had been larger with a higher statistical power.

Despite both this, a possible healthy-worker effect, risk of exposure misclassification, and the crude nicotine-exposure information, our exposure index still detected health effects from long-term occupational exposure, previously only described in case studies and after serious incidents. All in all, our observed effects are relatively modest, and biologically relevant, and the exposure index thus seems sufficiently robust (Austigard et al. 2018; Heldal et al. 2019). Emphasizing exposure peaks more than cumulative exposure also seem reasonable, as H_2_S does not accumulate in the body but is easily metabolized and excreted.

## Conclusion

We observe impaired bodily balance associated with long-term H_2_S exposure among smokers, when tested blindfolded. The results indicate that low-level occupational H_2_S exposure with exposure peaks in certain work operations may be hazardous to health and pinpoints the importance of minimizing and avoiding exposure peaks, also when H_2_S TWA measurements do not exceed an occupational exposure limit of 5 ppm. The outcome may be associated with H_2_S influencing cholinergic receptors or nerves in the brainstem or basal ganglia, but maintaining balance involves large parts of the CNS, and further studies regarding this is warranted. The study does not indicate comparable effects regarding reaction time, or fine motor coordination related to long-term exposure to H_2_S. Effects from short-term acute or sub-acute H_2_S exposure do not seem to conceal effects associated with the more long-term H_2_S index in this study. The test pattern does not indicate the presence of a hypothetical stimulating effect of low-dose H_2_S exposure.

## Supplementary Material

wxad051_suppl_Supplementary_Appendix_A

## Data Availability

Data will be deposited in the Norwegian Agency for Shared Services in Education and Research (Sikt) Research Data Archive (https://sikt.no/en).
