# Supplementary material for: A cross-sectional study of sensory-motor neuropsychological function among sewage plant and sewage net workers exposed to hydrogen sulphide when handling wastewater
Source: Ann Work Expo Health. 2023 Sep 23;67(9):1027–42. doi: 10.1093/annweh/wxad051 (PMC10683850; doi:10.1093/annweh/wxad051)
Supplement: wxad051_suppl_Supplementary_Appendix_A [file wxad051_suppl_supplementary_appendix_a.docx]

**Table A Appendix *.** Self-reported respiratory and nervous system symptoms among male sewage workers currently exposed to H_2_S at work (N=112), and the workplace control-group currently unexposed to H_2_S (N=26), and in four H_2_S-index subgroups of the total group (N=138).

|  | *Workers currently exposed and unexposed to H_2_S* | | | | *H_2_S-index – subgroups in the total group* | | | | | |
| --- | --- | --- | --- | --- | --- | --- | --- | --- | --- | --- |
|  | Currently exposed N=112 ^b^ | No current exposure N=26 |  |  | Group 1^a^  N=28 ^c^ | Group 2  N=71 | Group 3  N=20 | Group 4  N=19 |  |  |
|  | N (%) | N (%) | P ^d^ | RR (95% CI) ^e^ | N (%) | N (%) | N (%) | N (%) | P ^f^ |  |
| ***Respiratory symptoms*** |  |  |  |  |  |  |  |  |  |  |
| Nose | 67 (60.4) ^1^ | 15 (57.7) | *0.827* | 1.05 (0.73, 1.50) | 19 (67.9) | 37 (52.1) | 12 (63.2) ^6^ | 14 (73.7) | *0.753* |  |
| Cough | 62 (55.9) ^1^ | 13 (50.0) | *0.664* | 1.12 (0.74, 1.70) | **13 (46.4)** | 39 (54.9) | 8 (42.1) ^6^ | **15 (78.9)** | ***0.036*** |  |
| Cough last three months | 9 ( 8.1) ^1^ | 2 ( 7.7) | *1.000* | 1.05 (0.24, 4.59) | 1 ( 3.6) | 9 (12.7) | 0 ( 0.0) ^6^ | 1 ( 5.3) | *1.000* |  |
| Heavy breathing | 37 (33.6) ^2^ | 4 (15.4) | *0.095* | 2.19 (0.86, 5.59) | 8 (28.6) | 19 (27.1) ^4^ | 6 (31.6) ^6^ | 8 (42.1) | *0.366* |  |
| Wheezing | 44 (39.6) ^1^ | 10 (38.5) | *1.000* | 1.03 (0.60, 1.76) | 5 (17.9) | 36 (50.7) | 5 (26.3) ^6^ | 8 (42.1) | *0.099* |  |
| Airway symptoms | **37 (33.3) ^1^** | **3 (11.5)** | ***0.031*** | 2.89 (0.97, 8.65) | 5 (17.9) | 26 (36.6) | 4 (21.1) ^6^ | 5 (26.3) | *0.496* |  |
| Fever attacks | 40 (35.7) | 10 (38.5) | *0.823* | 0.93 (0.54, 1.60) | 9 (32.1) | 24 (33.8) | 11 (55.0) | 6 (31.6) | *1.000* |  |
| Nausea/diarrhea | 48 (42.9) | 10 (38.5) | *0.826* | 1.11 (0.66, 1.90) | 12 (42.9) | 31 (43.7) | 11 (55.0) | 4 (21.1) | *0.209* |  |
| ***Nervous system symptoms*** |  |  |  |  |  |  |  |  |  |  |
| Tired | 53 (48.2) ^2^ | 10 (38.5) | *0.392* | 1.25 (0.74, 2.11) | 10 (35.7) | 33 (47.1) ^4^ | 12 (63.2) ^6^ | 8 (42.1) | *0.763* |  |
| Palpitations | 10 ( 9.1) ^2^ | 0 ( 0.0) | *0.208* | - | 1 ( 3.6) | 5 ( 7.1) ^4^ | 2 (10.5) ^6^ | 2 (10.5) | *0.557* |  |
| Irritation | 15 (13.6) ^2^ | 1 ( 3.8) | *0.307* | 3.55 (0.49, 26.64) | 0 ( 0.0) | 10 (14.3) ^4^ | 3 (15.8) ^6^ | 3 (15.8) | *0.060* |  |
| Headache | 20 (18.3) ^3^ | 3 (11.5) | *0.565* | 1.59 (0.51, 4.95) | 3 (10.7) | 10 (14.5) ^5^ | 6 (31.6) ^6^ | 4 (21.1) | *0.417* |  |
| Forgetfulness | 54 (49.1) ^2^ | 9 (34.6) | *0.198* | 1.42 (0.81, 2.49) | 13 (46.4) | 35 (50.0) ^4^ | 9 (47.4) ^6^ | 6 (31.6) | *0.374* |  |
| Concentration difficulties | 36 (32.7) ^2^ | 7 (26.9) | *0.645* | 1.22 (0.61, 2.42) | 8 (28.6) | 26 (37.1) ^4^ | 5 (26.3) ^6^ | 4 (21.1) | *0.737* |  |
| Sweating | 15 (13.6) ^2^ | 3 (11.5) | *1.000* | 1.18 (0.37, 3.78) | 3 (10.7) | 10 (14.3) ^4^ | 0 ( 0.0) ^6^ | 5 (26.3) | *0.240* |  |
| Chest pressure | 18 (16.4) ^2^ | 2 ( 7.7) | *0.364* | 2.13 (0.53, 8.60) | 2 ( 7.1) | 10 (14.3) ^4^ | 4 (21.1) ^6^ | 4 (21.1) | *0.204* |  |
| Dizziness | 9 ( 8.2) ^2^ | 1 ( 3.8) | *0.687* | 2.13 (0.87, 16.06) | 2 ( 7.1) | 4 ( 5.7) ^4^ | 4 (21.1) ^6^ | 0 ( 0.0) | *0.508* |  |
| Hand tremor | 18 (16.4) ^2^ | 2 ( 7.7) | *0.364* | 2.13 (0.53, 8.60) | 7 (25.0) | 10 (14.3) ^4^ | 1 ( 5.3) ^6^ | 2 (10.5) | *0.278* |  |

^a^  H_2_S-index subgroup values: Group 1 (lowest): 0.54 - 1.39; Group 2: 3.08 - 4.40; Group 3: 6.29 - 7.29; Group 4 (highest): 9.25 - 10.16

^b^  Missing N: ^1^ N=111 ^2^ N=110 ^3^ N=109 ^4^ N=70 ^5^ N=69 ^6^ N=19

^c^  Distribution of currently H_2_S-exposed vs. currently unexposed workers in each subgroup: Group 1: N=24 vs. 4; Group 2:N=55 vs. 16; Group 3: N=16 vs. 4; Group 4: N=17 vs. 2.

^d^ Chi-square test, Fishers exact test (2-sided), H_2_S-exposed vs. workplace control group of currently unexposed workers.

^e^ Reference group is workplace control group of workers currently unexposed to H_2_S (N=26).

^f^ Chi-square test, Fishers exact test (2-sided), Group 1 vs. Group 4
